# Bleeding Outcomes After Dental Extraction in Patients Under Direct-Acting Oral Anticoagulants vs. Vitamin K Antagonists: A Systematic Review and Meta-Analysis

**DOI:** 10.3389/fphar.2021.702057

**Published:** 2021-10-28

**Authors:** Wenbing Hua, Zhengmei Huang, Zhuoli Huang

**Affiliations:** ^1^ Department of Stomatoogy, Shanghai PuDong Guangming Hospital of Traditional Chinese Medicine, Shanghai, China; ^2^ Department of Stomatoogy, Renji Hospital, School of Medcine, Shanghai Jiao Tong Universty, Shanghai, China; ^3^ Department of Implantology, Shanghai Ninth People’s Hospital, Shanghai Jiao Tong University School of Medicine; College of Stomatology, Shanghai Jiao Tong University; National Center for Stomatology; National Clinical Research Center for Oral Diseases; Shanghai Key Laboratory of Stomatology, Shanghai, China

**Keywords:** novel oral anticoagulants, warfarin, hemorrhage, oral surgery, anticoagulation

## Abstract

**Background:** The current systematic review aimed to compare bleeding outcomes in dental extraction patients receiving uninterrupted Direct-acting oral anticoagulant (DOAC) or Vitamin K antagonists (VKAs) for various systemic diseases.

**Methods:** PubMed, Embase, ScienceDirect, CENTRAL, and Google Scholar databases were searched for randomized controlled trials, controlled clinical trials, prospective and retrospective cohort studies, and case control studies, conducted on adult patients undergoing dental extraction under uninterrupted DOAC or VKAs therapy and reporting bleeding outcomes. The search was conducted up to March 31, 2021. We pooled data to calculate risk ratios (RR) with 95% confidence intervals (CI) in a random-effects model.

**Results:** Eight studies comparing 539 patients on DOAC therapy and 574 patients on VKAs were included. Meta-analysis indicated a statistically significant lower bleeding risk in patients under DOAC therapy (RR 0.68 95% CI 0.49, 0.95 I^2^ = 0%). However, on sensitivity analysis, the results were statistically non-significant after exclusion of any of the included studies. On pooled analysis of limited number of studies, we found no statistically significant difference in the risk of bleeding between apixaban (RR 0.85 95% CI 0.45, 1.60 I^2^ = 0%), rivaroxaban (RR 0.95 95% CI 0.36, 2.48 I^2^ = 45%), dabigatran (RR 0.49 95% CI 0.19, 1.28 I^2^ = 5%), edoxaban (RR 0.41 95% CI 0.13, 1.27 I^2^ = 0%) and VKAs.

**Conclusion:** The results of the first review comparing bleeding outcomes after dental extraction in patients on uninterrupted DOAC or VKA therapy indicates that patients on DOAC may have a reduced risk of hemorrhage. Current evidence is of very low-quality and should be interpreted with caution. Data on individual DOAC is scarce and at this point, the difference in the risk of bleeding between these drugs cannot be elucidated. Further studies with a large sample size shall supplement our conclusion.

## Introduction

Owing to rapid technological advancements and accessibility to healthcare, life expectancy has increased with a corresponding increase in the elderly population across the globe. Indeed, according to the National Institute of Health, United States around 8.5% of the world’s population is above 65 years of age and the figure is expected to jump to 17% by 2050 [National Institutes of Health (NIH), 2016]. As a result, the number of elderly patients requiring dental treatment is also expected to rise (Elani et al., 2018). Concomitant comorbidities requiring multiple drugs are extremely common in older adults. Treatment plans in these patients must therefore take into account any procedure-related adverse effects of such medications (Natarajan et al., 2019).

Antiplatelet and anticoagulant drugs are routinely prescribed after myocardial infarction, percutaneous coronary interventions, atrial fibrillation, joint arthroplasties, deep vein thrombosis, or pulmonary embolism to reduce the risk of systemic thromboembolism (Mega and Simon, 2015; Kapil et al., 2017). Over several decades, drugs like warfarin, acenocoumarol, phenprocoumon are collectively known as Vitamin K antagonists (VKAs) have been the primary drugs prescribed when anticoagulation is required (Malhotra et al., 2019). However, in recent times direct-acting oral anticoagulant (DOAC) drugs like apixaban, rivaroxaban, and dabigatran have achieved widespread adoption for preventing thromboembolism (Marzec et al., 2017; Raparelli et al., 2017). One reason for this change is that DOAC is thought to have a better safety profile with a more predictable anticoagulant action as compared to VKAs (Marzec et al., 2017; Raparelli et al., 2017).

While prescribing anticoagulants for any systemic illness, clinicians need to maintain a fine balance between the efficacy of the drug i.e., reducing the risk of thromboembolism, and safety of the therapy i.e., not increasing the risk of bleeding (Gu et al., 2019). As expected, any invasive procedures like dental extractions can become complicated if patients on anticoagulants are not efficiently managed in the perioperative period. Most practitioners are therefore hesitant to recommend any minor oral surgical procedure in patients under anticoagulant drugs (Ghantous and Ferneini, 2016). Over the years, much research has been conducted on whether uninterrupted anticoagulant use increases the risk of bleeding after dental extractions and most evidence suggests that uncomplicated extractions may be safely performed if adequate hemostatic measures are taken intraoperatively (Shi et al., 2017; De Andrade et al., 2019). Indeed, a recent meta-analysis of randomized controlled trials by De Andrade et al. (2019) has shown that the risk of bleeding after dental surgery does not change with the discontinuation of anticoagulants. However, it is still unclear if patients on a different class of anticoagulants have different bleeding tendencies (Doganay et al., 2018; Martínez-Moreno et al., 2021). The risk of bleeding in patients on DOAC or VKAs has been controversial with one meta-analysis (Brunetti et al., 2020) indicating no difference between the two groups while other studies suggest that risk of gastrointestinal bleeding may be increased with DOAC (Raschi et al., 2016, 2019).

With the increase in the utilization of DOAC in clinical practice (Raparelli et al., 2017), dental practitioners need to know the comparative bleeding tendency between newer DOACs and older VKAs. A few studies in the recent past have attempted to clarify evidence on this subject, however, with a limited sample size (Mauprivez et al., 2016; Lababidi et al., 2018). Thus, we hereby aimed to collate all available data to understand the difference in bleeding outcomes after dental extraction in patients on DOAC vs. VKAs. We believe the results of our review would help clinicians make informed decisions and better understand the risk of hemorrhage while performing a simple procedure like dental extraction in these patients.

## Materials and Methods

We adopted the guidelines of the PRISMA statement (Preferred Reporting Items for Systematic Reviews and Meta-analyses) (Page et al., 2021) for this systematic review and meta-analysis. Since publicly available databases were used for the analysis, institutional ethical approval was not required. The research question to be answered was: Is there a difference in bleeding outcomes after dental extraction in patients under uninterrupted DOAC or VKAs?

### Eligibility Criteria

The inclusion criteria of this review were framed according to the PICOS (Population, Intervention, Comparison, Outcome, and Study design) guidelines. Details are as follows:

1. Studies on a Population of adult patients (> 18 years) undergoing any kind of dental extraction.
2. The study was to include patients under uninterrupted DOAC therapy (Intervention) as one arm.
3. And patients under uninterrupted VKAs therapy in the other arm (Comparison).
4. Outcome to be assessed was bleeding episodes post-extraction reported either by the patient or on follow-up examination by the health-care professional. There was no minimum follow-up period required for inclusion.
5. Study designs included were randomized controlled trials, controlled clinical trials, prospective or retrospective cohort studies, and case-control studies.

The following studies were excluded: 1) Studies not reporting separate data for patients on DOAC and VKAs. 2) Studies interrupting the use of anticoagulants in the perioperative period. 3) Studies not reporting relevant outcomes. 4) Studies on other minor oral surgical procedures. 5) Review articles and non-English language studies. If two or more studies were found to report a duplicate or overlapping data, the study with the larger sample size was to be included. Studies including patients on dual antiplatelet and anticoagulant therapy were not excluded.

### Search Strategy

In consultation with a medical librarian, we searched PubMed, Embase, ScienceDirect, and CENTRAL databases to look for eligible studies. Also, Google Scholar was searched for the first 100 results of each query. All databases were screened from inception to March 31, 2021. The search was conducted by two reviewers independent of each other (Zhe. H and Zhu. H). Keywords used in different combinations were: “dental extraction,” “oral surgery,” “tooth extraction,” “direct oral anticoagulant,” “anticoagulant,” “vitamin K antagonist,” “warfarin,” “dabigatran,” “apixaban,” “rivaroxaban,” and “edoxaban.” Details of the search queries are presented in Supplementary Table S1. An adaptation of this search queries were used for all databases. Every search result was evaluated by the two reviewers independently. The initial screening was by the titles and abstracts of the searched articles. Relevant publications were selected for full-text review. Studies were then assessed based on the predefined eligibility criteria and the article satisfying all the criteria was included in this study. Any disagreements in the study selection process were resolved by discussion with the third reviewer (WH). To avoid any missed studies, the bibliography of included studies and recent reviews (Yang et al., 2016; Shi et al., 2017; De Andrade et al., 2019; Ockerman et al., 2020) on the topic were hand searched for any additional references.

### Data Extraction and Risk of Bias Assessment

We prepared a data extraction form at the beginning of the review to extract relevant details from the studies. Author details, year of publication, study location, study type, sample size, demographic details of the sample, drugs compared, number and type of extractions carried out, International Normalization Ratio (INR), use of any concomitant anti-platelet drugs, use of hemostatic measures post-extraction, the definition of bleeding, and number of bleeding episodes were extracted. Data were extracted in duplicate by two authors and checked for correctness. Surgical extractions were defined as transalveolar extractions involving raising of mucoperiosteal flaps with removal of bone.

The methodological quality of included studies was assessed using the ROBINS-1 tool (Sterne et al., 2016). This too was carried out in duplicate and independently by two study investigators (Zhe. H and Zhu. H). Studies were assessed for the following domains: Bias related to confounding, selection of participants, classification of interventions, departure from intended intervention, missing data, measurement of outcomes, and selection of overall results. Studies were marked as low, moderate, serious or critical risk of bias. Any discrepancies were resolved in consultation with the third reviewer (WH). The certainty of the evidence was assessed using the Grading of Recommendations Assessment, Development, and Evaluation (GRADE) tool using the GRADEpro GDT software [GRADEpro Guideline Development Tool. McMaster University, 2020 (developed by Evidence Prime, Inc.)].

### Statistical Analysis

“Review Manager” [RevMan, version 5.3; Nordic Cochrane Centre (Cochrane Collaboration), Copenhagen, Denmark; 2014] was used for the meta-analysis. Since the outcome data were dichotomous, we pooled it to calculate risk ratios (RR) with 95% confidence intervals (CI). A random-effects model was preferred for the meta-analysis considering the fact that there would be methodological variations in the included studies. The I^2^ statistic was used to assess inter-study heterogeneity. According to Cochrane handbook, I^2^ values of 0–40% may not be important, values of 30–60% represent moderate heterogeneity, values of 50–90% represent substantial heterogeneity and more than 75% represent considerable heterogeneity (Higgins et al., 2019). Visual inspection of funnel plot was carried out to assess publication bias. A sensitivity analysis was conducted to check the effect of each study on the final RR. We excluded data of every study sequentially to recalculate the effect size. Results were presented in a tabular format. A subgroup analysis was also conducted for studies including patients on antiplatelet therapy and those not including such patients. We also performed separate analysis to compare specific DOAC with VKAs.

## Results

### Study Details

**FIGURE 1:**
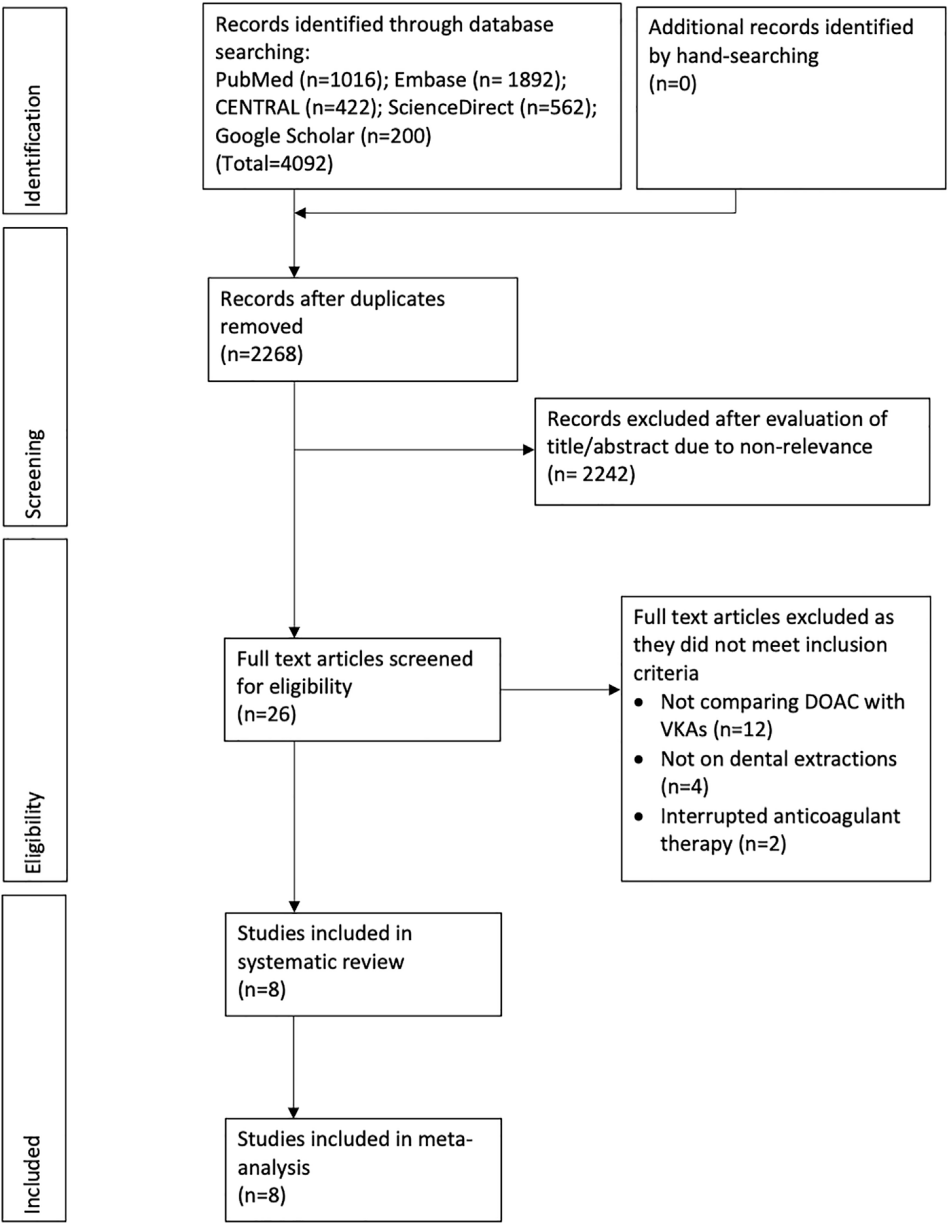
Study flow chart.

The study flow chart is presented in Figure 1. Finally, eight studies fulfilled the inclusion criteria and were included in this review (Mauprivez et al., 2016; Caliskan et al., 2017; Yagyuu et al., 2017; Lababidi et al., 2018; Berton et al., 2019; Yoshikawa et al., 2019; Brennan et al., 2020; Inokoshi et al., 2021). Characteristics of included studies are presented in Table 1. Five of the included studies were carried out prospectively while the remaining were retrospective analyses. The sample size of the DOAC arm varied from 21 to 138 patients while the VKAs arm varied from 20 to 248 patients. Only two studies (Caliskan et al., 2017; Berton et al., 2019) carried out single tooth extraction while others carried out single and multiple extractions. Surgical extraction consisting of elevation of the mucoperiosteal flap with bone cutting was carried out in four studies (Mauprivez et al., 2016; Lababidi et al., 2018; Yoshikawa et al., 2019; Brennan et al., 2020). However, the overall percentage of surgical extractions was <20% in all studies. Concomitant use of antiplatelet drugs was seen in five studies (Yagyuu et al., 2017; Lababidi et al., 2018; Yoshikawa et al., 2019; Brennan et al., 2020; Inokoshi et al., 2021). In three of these studies (Yagyuu et al., 2017; Lababidi et al., 2018; Yoshikawa et al., 2019), the use of antiplatelet drugs was significantly lower in the DOAC arm. INR range for the VKA group varied in the included studies. The use of local hemostatic measures consisting of placement of a hemostatic agent like oxidized cellulose and suturing was reported by a majority of included studies.

**TABLE 1:** Details of included studies.

Study	Location	Study type	Indication for anticoagulation	Study groups	Sample size	Mean age (years)	Male gender (%)	Number of extractions	Surgical extractions (%)	AP drugs (%)	INR	Intra-operative hemostasis technique
Inokoshi et al. (2021)	Japan	Retrospective	AF, IHD, DVT, PE, VHD, arrhythmias	DOAC	138	80 ± 6	49.3	1.49 ± NR	0	NR	NR	Hemostatic agent, suturing
				Warfarin	98	78.9 ± 6.5	49	1.65 ± NR	0		<3.5	
Brennan et al. (2019)	Australia	Prospective	NR	DOAC	86	73 (67–78)^b^	63	1 (1–2)^b^	17	11	NR	Suturing, oxidized cellulose, and pressure pack. Tranexamic acid pack in case of bleeding after 60 min
				Warfarin	21	71 (62–79)	96	2 (1–3)	10	0	2–4	
Berton et al. (2019)	Italy	Prospective	AF, DVT, PE, Stroke	DOAC	65	76 ± 9.2	52.3	1	0	0	NR	Oxidized cellulose sponges or tranexamic acid pack
				Warfarin	65	76 ± 7.7	47.7	1	0	0	2–3	
Yoshikawa et al. (2019)	Japan	Prospective	AF, IHD, DVT, VHD, stroke	DOAC	119	74.6 ± 10.1	68.9	Multiple:55.5%	2.1	15.1	NR	Suturing, oxidized cellulose
				Warfarin	248	71.6 ± 10.1	65.7	Multiple: 53.4%	4.4	33.5	2.08 ± 0.47	
Lababidi et al. (2018)	Australia	Retrospective	AF, VHD, DVT, PE	DOAC	38	72 ± 2	46.5	Multiple:39.6%	11.3	4.7	NR	Hemostatic agent, suturing, tranexamic acid mouthwash
				Warfarin	50	71.1.5	52	Multiple: 33.9%	10.1	24	2.2–4^a^	
Yagyuu et al. (2017)	Japan	Retrospective	NR	DOAC	41	72.3 ± 7.1	52.8	Total: 72	0	5.6	1.17 ± 0.12	NR
				Warfarin	50	73.7 ± 15.6	63	Total: 100	0	15	1.63 ± 0.39	
Caliskan et al. (2017)	Turkey	Prospective	AF, VHD, DVT, PE, IHD, stroke	DOAC	21	60.8 ± 11.8	57	1	0	0	1.81 ± 1.3	Suturing, oxidized cellulose
				Warfarin	22	60.7 ± 10.4	73	1	0	0	2.33 ± 0.5	
Mauprivez et al. (2016)	France	Prospective	AF, DVT, PE, IHD, stroke	DOAC	31	70.3 ± 2.1	45.2	Multiple:61.3%	12.3	0	NR	Gelatin sponge, suturing
				Warfarin	20	70.6 ± 2.8	55	Multiple: 80%	18.9	0	2.28 ± 0.1	

NR, not reported; NOS, Newcastle-Ottawa Scale; VKA, vitamin K inhibitors; AP, antiplatelet drugs; AF atrial fibrillation; IHD, ischemic heart disease; DVT, deep vein thrombosis; PE, pulmonary embolism; VHD, valvular heart disease.

a 8 patients had INR <2.2.

b Median (Interquartile range).

### Bleeding Outcome

**FIGURE 2:**
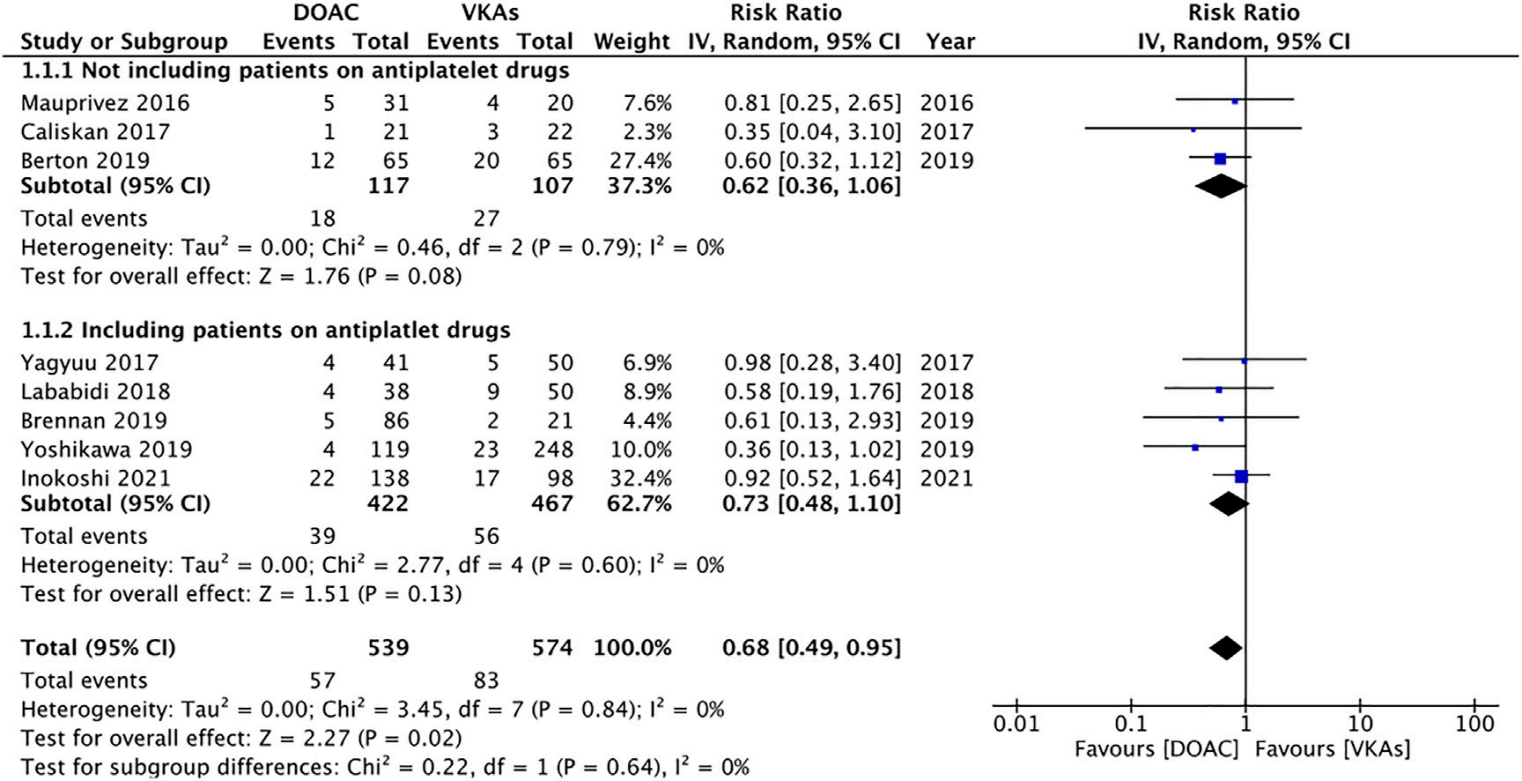
Meta-analysis of bleeding outcomes between patients under DOAC vs. VKAs with subgroup analysis based on inclusion of patients on antiplatelet drugs.

**FIGURE 3:**
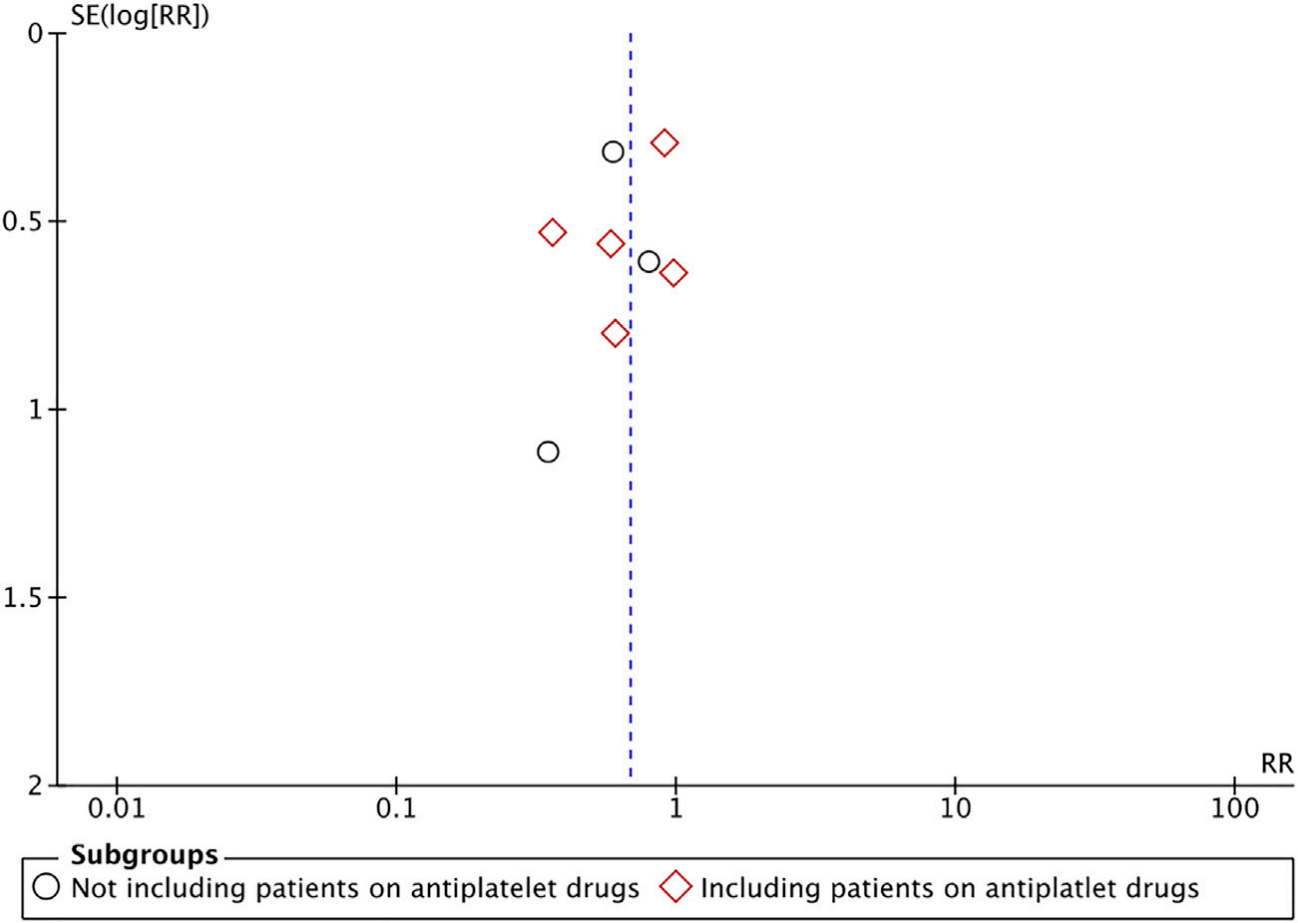
Funnel plot for assessing publication bias.

The definition of bleeding had minor variations in the included studies. Details are presented in Table 2. Overall it may be subsumed that bleeding was defined as any oozing or hemorrhage that required some intervention for it to be controlled. Meta-analysis of all eight studies with 539 patients in the DOAC group and 574 patients in the VKAs group indicated a statistically lower bleeding risk in patients under DOAC therapy (RR 0.68 95% CI 0.49, 0.95 I^2^ = 0%) (Figure 2). There was no evidence of publication bias on the funnel plot (Figure 3). The certainty of evidence based on GRADE was “very low” (Supplementary Table S2). On sensitivity analysis, the results were indicated a statistically non-significant difference between the two groups after exclusion of any of the included studies, albeit with a tendency of lower bleeding risk with DOAC (Table 3). In addition to the sensitivity analysis, we also conducted a subgroup analysis based on the inclusion of patients on antiplatelet drugs. Our analysis revealed a non-significant but lower tendency of bleeding in patients on DOAC in studies including patients on antiplatelet drugs (RR 0.73 95% CI 0.48, 1.10 I^2^ = 0%) as well as those studies not including patients on antiplatelets (RR 0.62 95% CI 0.36, 1.06 I^2^ = 0%) (Figure 2).

**TABLE 2:** Definition of bleeding outcome in included studies.

Study	Definition
Inokoshi et al. (2021)	Oozing or marked hemorrhaging persisting from 24 h to 7 days after dental extraction that required treatment to arrest bleeding
Brennan et al. (2019)	Bleeding that required medical intervention by a health care provider including oral anticoagulant discontinuation, bleeding that led to hospitalization or increased level of care without requiring surgical intervention, and bleeding that led to face-to-face evaluation
Berton et al. (2019)	Bleeding that required compression packs, pharmacological intervention or surgical intervention
Yoshikawa et al. (2019)	Oozing or marked haemorrhage that could not be stopped by wound compression with gauze, and haemostasis that required medical intervention such as haematoma removal, curettage, suturing, or splint placement
Lababidi et al. (2018)	Bleeding managed with direct measures such as pressure at home or by a clinician
Yagyuu et al. (2017)	Bleeding that could not be stopped by biting down on gauze and that required medical treatment between 30 min and 7 days after the tooth extraction
Caliskan et al. (2017)	Bleeding managed with gauze pads, hemostatic agents or bleeding requiring hospitalization
Mauprivez et al. (2016)	Persistent oozing or marked hemorrhage over 20 min after tooth extraction despite local hemostasis procedures or all bleeding episode occurring during the first postoperative week

**TABLE 3:** Results of sensitivity analysis.

Excluded study	Resultant effect size (risk ratios)
Inokoshi et al. (2021)	0.65 (95% CI 0.42, 1.00 I^2^ = 0%)
Brennan et al. (2019)	0.74 (95% CI 0.52, 1.06 I^2^ = 0%)
Berton et al. (2019)	0.80 (95% CI 0.53, 1.21 I^2^ = 0%)
Yoshikawa et al. (2019)	0.73 (95% CI 0.52, 1.04 I^2^ = 0%)
Lababidi et al. (2018)	0.75 (95% CI 0.52, 1.08 I^2^ = 0%)
Yagyuu et al. (2017)	0.72 (95% CI 0.50, 1.03 I^2^ = 0%)
Caliskan et al. (2017)	0.75 (95% CI 0.53, 1.06 I^2^ = 0%)
Mauprivez et al. (2016)	0.73 (95% CI 0.51, 1.04 I^2^ = 0%)

CI, confidence intervals.

**FIGURE 4:**
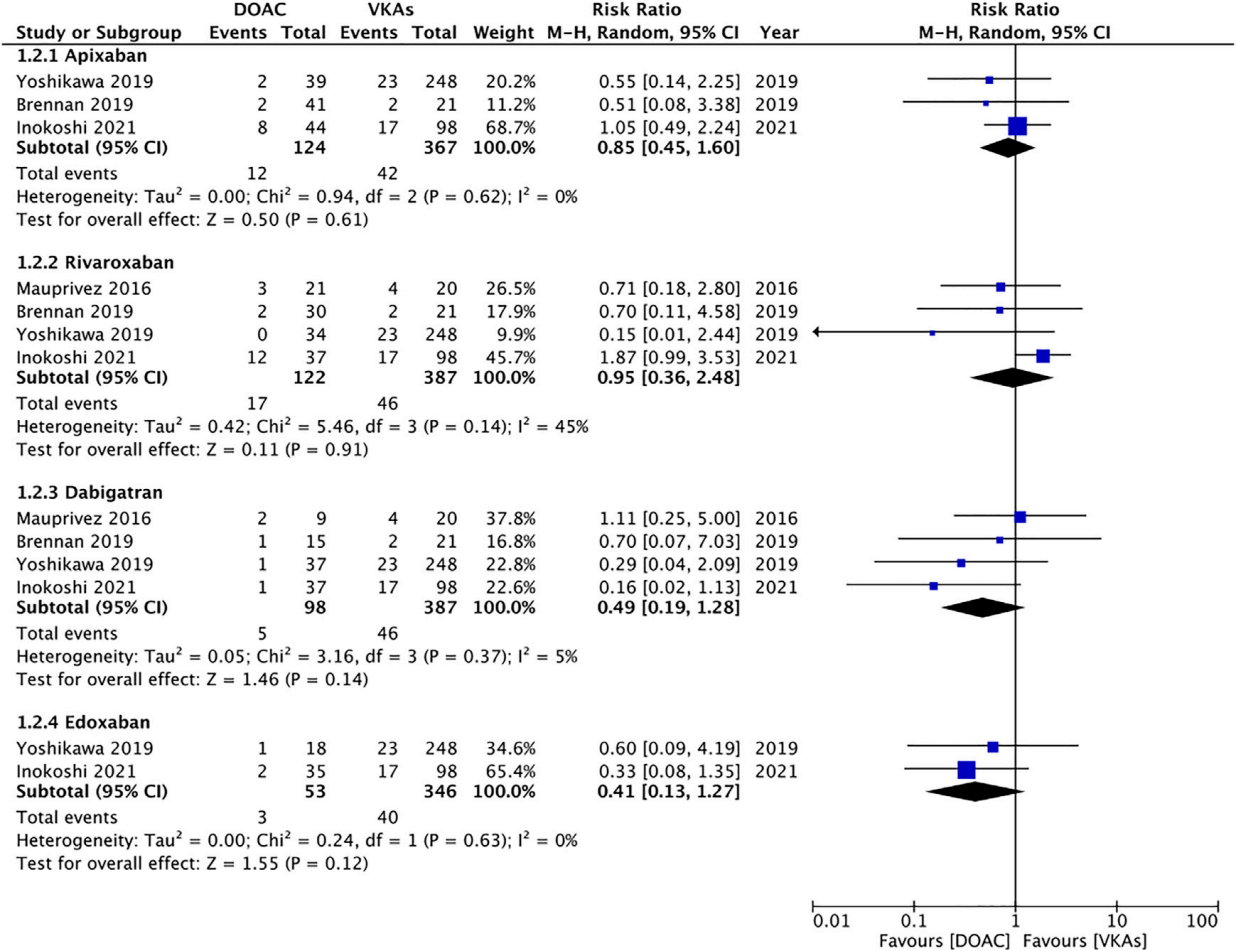
Meta-analysis of bleeding outcomes between patients under different DOAC vs. VKAs.

Data for individual DOAC were reported by limited number of studies. On pooled analysis, we found no statistically significant difference in the risk of bleeding between apixaban (RR 0.85 95% CI 0.45, 1.60 I^2^ = 0% *p* = 0.61), rivaroxaban (RR 0.95 95% CI 0.36, 2.48 I^2^ = 45%), dabigatran (RR 0.49 95% CI 0.19, 1.28 I^2^ = 5%), endoxaban (RR 0.41 95% CI 0.13, 1.27 I^2^ = 0%) and VKAs (Figure 4). The certainty of evidence based on GRADE was “very low” (Supplementary Table S2).

### Risk of Bias Assessment

Authors judgement of the risk of bias amongst studies based on ROBIN-1 tool is presented in Table 4. One study had low risk of bias (Berton et al., 2019), four had moderate risk of bias (Mauprivez et al., 2016; Caliskan et al., 2017; Brennan et al., 2020; Inokoshi et al., 2021) and three had serious risk of bias (Yagyuu et al., 2017; Lababidi et al., 2018; Yoshikawa et al., 2019).

**TABLE 4:** Risk of bias analysis for included studies using ROBINS-1 tool.

Study	Bias due to confounding	Bias in selection of participants	Bias in classification of interventions	Bias due to departures from intended interventions	Bias due to missing data	Bias in measurement of outcomes	Bias in selection of reports results	Overall ROBINS-1 judgment
Inokoshi et al. (2021)	Moderate	Moderate	Low	Low	Low	Moderate	Low	Moderate
Brennan et al. (2019)	Moderate	Low	Low	Low	Low	Low	Low	Moderate
Berton et al. (2019)	Low	Low	Low	Low	Low	Low	Low	Low
Yoshikawa et al. (2019)	Serious	Low	Low	Low	Low	Low	Low	Serious
Lababidi et al. (2018)	Serious	Moderate	Low	Low	Low	Moderate	Low	Serious
Yagyuu et al. (2017)	Serious	Moderate	Low	Low	Low	Low	Low	Serious
Caliskan et al. (2017)	Low	Low	Low	Low	Low	Moderate	Low	Moderate
Mauprivez et al. (2016)	Moderate	Low	Low	Low	Low	Moderate	Low	Moderate

## Discussion

Anticoagulation therapy is known to offer an important therapeutic advantage by reducing the risk of thromboembolism in many systemic diseases (Mega and Simon, 2015; Kapil et al., 2017). VKAs have been the gold-standard drugs for systemic anticoagulation for several years. However, an important disadvantage with their use is the need for frequent dose adjustments to maintain the therapeutic range of INR. Furthermore, VKAs have multiple interactions with foods and other medications, slow onset of action necessitating overlapping use of heparins, and increased risk of hemorrhagic events (Zolfaghari et al., 2014; Ntaios et al., 2017). The development of DOAC, which acts directly on a single coagulation factor, has more or less overcome these limitations. They have a rapid onset of action, limited drug or food interactions, do not need therapeutic monitoring, and with overall superior efficacy and safety profile (Almutairi et al., 2017; Ntaios et al., 2017). Indeed, in a meta-analysis of seven randomized controlled trials (RCTs), Almutairi et al. (2017) have demonstrated a 32–69% reduced risk of major hemorrhage with DOAC as compared to VKA. However, there is a lack of clarity on the risk of hemorrhage after minor surgical procedures like dental extraction between the two classes of drugs. Despite the overall better profile of DOAC, have been concerns amongst clinicians regarding the reversibility of these agents in case of severe bleeding. While reversal agents like andexanet alfa have recently been developed, the cost and availability is still a limiting factor (Frontera et al., 2020). Secondly, clinicians may monitor INR in patients under VKAs to gauge the risk of bleeding, but no such tool is available for DOAC (Favaloro et al., 2017).

It is now well-established that uncomplicated dental extractions can be safely carried out without interruption of VKAs therapy provided INR is maintained <3.5 and local hemostatic measures are performed to control bleeding (Nematullah et al., 2009). However, no clear guidelines exist on the perioperative management of patients receiving DOAC (Brennan et al., 2019). Recommendations range from the continuation of DOAC during dental extractions to omitting one or two doses of the drug before the procedure (Brennan et al., 2019). A recent survey by Precht et al. (2019) has demonstrated 94% of dental practitioners continue with VKAs during single tooth extractions but 62% of them interrupt DOAC therapy. Given such ambiguity, the results of our review present some clarity on the risk of hemorrhage with uninterrupted DOAC as compared to uninterrupted VKAs after dental extractions. We found a statistically significant reduced risk of hemorrhage with DOAC as compared to VKAs. Individually, none of the included studies noted a significant difference and all of them concluded that patients on DOACs have a similar bleeding tendency as compared to VKAs. We believe the limited sample size may have contributed to the individual non-significant results and the pooled analysis comparing data of >1,000 patients significantly raised the power of the analysis. This was also noted in the sensitivity analysis with the effect size being non-significant on the exclusion of any included study. However, it should also be noted that in the overall analysis, the upper limit of the 95% CI was 0.95, which is very close to 1, indicating no difference. The low precision of the estimates and the observational nature of the studies, prompted us to downgrade the overall quality of evidence. Hence, at this point these results should be interpreted with caution and should be clarified by future studies.

The lower risk of hemorrhage with DOAC as compared to VKAs may be linked to the difference in the pharmacokinetics of the drugs. VKAs act by inhibition of several coagulation factors and provide a persistent anticoagulant effect without any diurnal variation owing to their long half-life ranging from 55 to 133 h. On the other hand, DOAC offers intermittent anticoagulation due to their shorter half-life of 7–17 h, and the peak drug concentration is reached at 1–4 h (Ieko et al., 2016). It is plausible that the time gap between the last dose of DOAC and dental extraction may have reduced the risk of hemorrhage in these patients. Secondly, the overall better safety profile of DOAC as compared to VKAs may have also contributed to this difference (Almutairi et al., 2017). However, due to the scarce literature available on dental procedures, it is also important to interpret our results with studies comparing bleeding risks between DOAC and VKAs for other surgical procedures. A recent meta-analysis of RCTs has demonstrated significantly lower risk of major bleeding with DOAC as compared to VKAs in a patient undergoing catheter ablation for atrial fibrillation (Brunetti et al., 2020). On the other hand, literature also suggests that while intracranial bleeding is undoubtedly significantly reduced with DOACs, the risk of gastrointestinal bleeding can be increased, especially for rivaroxaban (Raschi et al., 2016; Raschi et al.,2019). It should also be noted that in our meta-analysis on rivaroxaban, the study of Inokoshi et al. (2021) demonstrated a non-significant increased risk of bleeding with the DOAC (RR: 1.87 95% Ci: 0.99, 3.53) and there was high heterogeneity in the analysis. Thus, evidence on bleeding tendencies with DOAC vs. VKAs is still upcoming and contradictory which needs further investigation.

Research indicates that dual antiplatelet therapy is associated with a significantly higher incidence of postoperative bleeding as compared to single antiplatelet therapy (Ockerman et al., 2020). Along similar lines, the use of antiplatelets with anticoagulants would lead to higher bleeding episodes. In our review, five studies (Yagyuu et al., 2017; Lababidi et al., 2018; Yoshikawa et al., 2019; Brennan et al., 2020; Inokoshi et al., 2021) reported concomitant use of antiplatelet drugs in the study group. However, on subgroup analysis based on this variable, we still noted a non-significant but tendency of reduced bleeding with DOAC. We also attempted to explore the role of individual DOAC on bleeding outcomes but could include only limited studies in the meta-analysis due to lack of data. The small sample size of the sub-group analysis failed to demonstrate any statistically significant difference in the risk of bleeding between individual DOACs and VKAs.

Our review has some limitations. Foremost, not all studies were prospective analyses. Retrospective studies based on medical records have an inherent source of bias. Secondly, as expected there was methodological heterogeneity in the included studies. While the majority of studies included patients with INR of two to four in the VKA group, there were some variations. This could have influenced outcomes. Furthermore, the definition of outcomes was not exactly coherent. This may have caused underestimation or overestimation of bleeding outcomes. Also, not all studies were on single non-surgical tooth extraction. The degree of invasiveness of the procedure is an important variable affecting hemorrhagic episodes. Majority of the studies did not report HASBLED scores which have important implications for bleeding outcomes. Another important point to consider is that we could assess only postoperative bleeding outcomes in our analysis as data on intra-operative bleeding was poorly reported amongst the included studies. Thirdly, the number of included studies was not very high and the results were not stable on sensitivity analysis. Furthermore, due to limited data, we were unable to conduct a robust analysis for individual DOACs. Lastly, we were unable to prospectively register the protocol of our review on any online database due to time constraints. This is also a significant limitation of our study. Furthermore, our literature search was restricted to published and English language studies only. Despite finding no non-English language study relevant to the review during the literature search, we may have inadvertently missed some non-English language studies. Also, some unpublished work could also have been missed as such literature was not searched for.

Considering the fact that a large number of clinicians recommend interruption of DOAC for routine dental extractions due to apprehensions regarding bleeding and availability of reversal agents (Precht et al., 2019), our results presents important evidence for clinical practice. According to our results, despite the lack of specific hematological monitoring of DOAC, dental extractions carried out on individuals on continued DOAC therapy does not lead to excessive bleeding as compared to patients on VKAs. On the contrary, the low-quality evidence suggests that the risk of bleeding may be significantly lower in patients on DOAC. Therefore, clinicians should not be apprehensive of patients on DOAC therapy requiring dental extractions. The procedure should be performed without discontinuing the drugs, however, with appropriate local hemostatic measures. While the lowered risk of bleeding with DOACs should be confirmed with further large scale studies, general physicians should also take note of the reduced hemorrhagic tendency associated with DOAC and these drugs could be preferred over VKAs for prophylaxis against thromboembolisms.

To conclude, the results of the first review comparing bleeding outcomes after dental extraction in patients on uninterrupted DOAC or VKA therapy indicate that patients on DOAC have a reduced risk of hemorrhage. Current evidence is of low-quality and should be interpreted with caution. Data on individual DOAC is scarce and at this point, the difference in the risk of bleeding between these drugs cannot be elucidated. Further high-quality studies are needed to strengthen current evidence.
