# Supplementary material for: Bleeding Outcomes After Dental Extraction in Patients Under Direct-Acting Oral Anticoagulants vs. Vitamin K Antagonists: A Systematic Review and Meta-Analysis
Source: Front Pharmacol. 2021 Oct 28;12:702057. doi: 10.3389/fphar.2021.702057 (PMC8585494; doi:10.3389/fphar.2021.702057)
Supplement: Supplementary file 1 [file Table1.DOCX]

Supplementary Table 1: Search details and results of PubMed database

| **Query** | **Search Details** | **Results** |
| --- | --- | --- |
| ((oral surgery[Title/Abstract]) OR (oral surgical[Title/Abstract])) AND (warfarin) | ("oral surgery"[Title/Abstract] OR "oral surgical"[Title/Abstract]) AND ("warfarin"[MeSH Terms] OR "warfarin"[All Fields] OR "warfarin s"[All Fields] OR "warfarinization"[All Fields] OR "warfarinized"[All Fields] OR "warfarins"[All Fields]) | 52 |
| ((oral surgery[Title/Abstract]) OR (oral surgical[Title/Abstract])) AND (anticoagulant) | ("oral surgery"[Title/Abstract] OR "oral surgical"[Title/Abstract]) AND ("anticoagulants"[Pharmacological Action] OR "anticoagulants"[MeSH Terms] OR "anticoagulants"[All Fields] OR "anticoagulant"[All Fields] OR "anticoagulate"[All Fields] OR "anticoagulated"[All Fields] OR "anticoagulating"[All Fields] OR "anticoagulation"[All Fields] OR "anticoagulations"[All Fields] OR "anticoagulative"[All Fields]) | 189 |
| ((dental extraction) OR (tooth extraction)) AND (anticoagulant) | ("tooth extraction"[MeSH Terms] OR ("tooth"[All Fields] AND "extraction"[All Fields]) OR "tooth extraction"[All Fields] OR ("dental"[All Fields] AND "extraction"[All Fields]) OR "dental extraction"[All Fields] OR ("tooth extraction"[MeSH Terms] OR ("tooth"[All Fields] AND "extraction"[All Fields]) OR "tooth extraction"[All Fields])) AND ("anticoagulants"[Pharmacological Action] OR "anticoagulants"[MeSH Terms] OR "anticoagulants"[All Fields] OR "anticoagulant"[All Fields] OR "anticoagulate"[All Fields] OR "anticoagulated"[All Fields] OR "anticoagulating"[All Fields] OR "anticoagulation"[All Fields] OR "anticoagulations"[All Fields] OR "anticoagulative"[All Fields]) | 521 |
| ((dental extraction) OR (tooth extraction)) AND (edoxaban) | ("tooth extraction"[MeSH Terms] OR ("tooth"[All Fields] AND "extraction"[All Fields]) OR "tooth extraction"[All Fields] OR ("dental"[All Fields] AND "extraction"[All Fields]) OR "dental extraction"[All Fields] OR ("tooth extraction"[MeSH Terms] OR ("tooth"[All Fields] AND "extraction"[All Fields]) OR "tooth extraction"[All Fields])) AND ("edoxaban"[Supplementary Concept] OR "edoxaban"[All Fields]) | 2 |
| ((dental extraction) OR (tooth extraction)) AND (rivaroxaban) | ("tooth extraction"[MeSH Terms] OR ("tooth"[All Fields] AND "extraction"[All Fields]) OR "tooth extraction"[All Fields] OR ("dental"[All Fields] AND "extraction"[All Fields]) OR "dental extraction"[All Fields] OR ("tooth extraction"[MeSH Terms] OR ("tooth"[All Fields] AND "extraction"[All Fields]) OR "tooth extraction"[All Fields])) AND ("rivaroxaban"[MeSH Terms] OR "rivaroxaban"[All Fields]) | 13 |
| ((dental extraction) OR (tooth extraction)) AND (apixaban) | ("tooth extraction"[MeSH Terms] OR ("tooth"[All Fields] AND "extraction"[All Fields]) OR "tooth extraction"[All Fields] OR ("dental"[All Fields] AND "extraction"[All Fields]) OR "dental extraction"[All Fields] OR ("tooth extraction"[MeSH Terms] OR ("tooth"[All Fields] AND "extraction"[All Fields]) OR "tooth extraction"[All Fields])) AND ("apixaban"[Supplementary Concept] OR "apixaban"[All Fields] OR "apixaban s"[All Fields]) | 9 |
| ((dental extraction) OR (tooth extraction)) AND (dabigatran) | ("tooth extraction"[MeSH Terms] OR ("tooth"[All Fields] AND "extraction"[All Fields]) OR "tooth extraction"[All Fields] OR ("dental"[All Fields] AND "extraction"[All Fields]) OR "dental extraction"[All Fields] OR ("tooth extraction"[MeSH Terms] OR ("tooth"[All Fields] AND "extraction"[All Fields]) OR "tooth extraction"[All Fields])) AND ("dabigatran"[MeSH Terms] OR "dabigatran"[All Fields] OR "dabigatran s"[All Fields]) | 13 |
| ((dental extraction) OR (tooth extraction)) AND (warfarin) | ("tooth extraction"[MeSH Terms] OR ("tooth"[All Fields] AND "extraction"[All Fields]) OR "tooth extraction"[All Fields] OR ("dental"[All Fields] AND "extraction"[All Fields]) OR "dental extraction"[All Fields] OR ("tooth extraction"[MeSH Terms] OR ("tooth"[All Fields] AND "extraction"[All Fields]) OR "tooth extraction"[All Fields])) AND ("warfarin"[MeSH Terms] OR "warfarin"[All Fields] OR "warfarin s"[All Fields] OR "warfarinization"[All Fields] OR "warfarinized"[All Fields] OR "warfarins"[All Fields]) | 147 |
| ((dental extraction) OR (tooth extraction)) AND (vitamin K antagonist) | ("tooth extraction"[MeSH Terms] OR ("tooth"[All Fields] AND "extraction"[All Fields]) OR "tooth extraction"[All Fields] OR ("dental"[All Fields] AND "extraction"[All Fields]) OR "dental extraction"[All Fields] OR ("tooth extraction"[MeSH Terms] OR ("tooth"[All Fields] AND "extraction"[All Fields]) OR "tooth extraction"[All Fields])) AND (("vitamin k"[MeSH Terms] OR "vitamin k"[All Fields]) AND ("antagonist"[All Fields] OR "antagonists and inhibitors"[MeSH Subheading] OR ("antagonists"[All Fields] AND "inhibitors"[All Fields]) OR "antagonists and inhibitors"[All Fields] OR "antagonists"[All Fields])) | 35 |
| ((dental extraction) OR (tooth extraction)) AND (direct oral anticoagulant) | ("tooth extraction"[MeSH Terms] OR ("tooth"[All Fields] AND "extraction"[All Fields]) OR "tooth extraction"[All Fields] OR ("dental"[All Fields] AND "extraction"[All Fields]) OR "dental extraction"[All Fields] OR ("tooth extraction"[MeSH Terms] OR ("tooth"[All Fields] AND "extraction"[All Fields]) OR "tooth extraction"[All Fields])) AND (("direct"[All Fields] OR "directed"[All Fields] OR "directing"[All Fields] OR "direction"[All Fields] OR "directional"[All Fields] OR "directions"[All Fields] OR "directivities"[All Fields] OR "directivity"[All Fields] OR "directs"[All Fields]) AND ("mouth"[MeSH Terms] OR "mouth"[All Fields] OR "oral"[All Fields]) AND ("anticoagulants"[Pharmacological Action] OR "anticoagulants"[MeSH Terms] OR "anticoagulants"[All Fields] OR "anticoagulant"[All Fields] OR "anticoagulate"[All Fields] OR "anticoagulated"[All Fields] OR "anticoagulating"[All Fields] OR "anticoagulation"[All Fields] OR "anticoagulations"[All Fields] OR "anticoagulative"[All Fields])) | 35 |
