# Supplementary material for: Bleeding Outcomes After Dental Extraction in Patients Under Direct-Acting Oral Anticoagulants vs. Vitamin K Antagonists: A Systematic Review and Meta-Analysis
Source: Front Pharmacol. 2021 Oct 28;12:702057. doi: 10.3389/fphar.2021.702057 (PMC8585494; doi:10.3389/fphar.2021.702057)
Supplement: Supplementary file 2 [file Table2.DOCX]

| **Supplementary Table 2: GRADE assessment of evidence** | | | | | | | | | | | |
| --- | --- | --- | --- | --- | --- | --- | --- | --- | --- | --- | --- |
| **Certainty assessment** | | | | | | | **Summary of findings** | | | | |
| **Participants  (studies) Follow up** | **Risk of bias** | **Inconsistency** | **Indirectness** | **Imprecision** | **Publication bias** | **Overall certainty of evidence** | **Study event rates (%)** | | **Relative effect (95% CI)** | **Anticipated absolute effects** | |
|  |  |  |  |  |  |  | **With VKAs** | **With DOAC** |  | **Risk with VKAs** | **Risk difference with DOAC** |
| **Bleeding** | | | | | | | | | | | |
| 1113 (8 observational studies) | very serious ^a^ | not serious | not serious | serious ^b^ | none | ⨁◯◯◯ VERY LOW | 83/574 (14.5%) | 57/539 (10.6%) | **RR 0.68** (0.49 to 0.95) | 145 per 1,000 | **46 fewer per 1,000** (from 74 fewer to 7 fewer) |
| **Bleeding - Apixaban** | | | | | | | | | | | |
| 491 (3 observational studies) | very serious ^c^ | not serious | not serious | not serious | none | ⨁◯◯◯ VERY LOW | 42/367 (11.4%) | 12/124 (9.7%) | **RR 0.85** (0.45 to 1.60) | 114 per 1,000 | **17 fewer per 1,000** (from 63 fewer to 69 more) |
| **Bleeding - Rivaroxaban** | | | | | | | | | | | |
| 509 (4 observational studies) | very serious ^c^ | serious ^d^ | not serious | not serious | none | ⨁◯◯◯ VERY LOW | 46/387 (11.9%) | 17/122 (13.9%) | **RR 0.95** (0.36 to 2.48) | 119 per 1,000 | **6 fewer per 1,000** (from 76 fewer to 176 more) |
| **Bleeding - Dabigatran** | | | | | | | | | | | |
| 485 (4 observational studies) | very serious ^c^ | not serious | not serious | not serious | none | ⨁◯◯◯ VERY LOW | 46/387 (11.9%) | 5/98 (5.1%) | **RR 0.49** (0.19 to 1.28) | 119 per 1,000 | **61 fewer per 1,000** (from 96 fewer to 33 more) |
| **Bleeding - Edoxaban** | | | | | | | | | | | |
| 399 (2 observational studies) | very serious ^c^ | not serious | not serious | not serious | none | ⨁◯◯◯ VERY LOW | 40/346 (11.6%) | 3/53 (5.7%) | **RR 0.41** (0.13 to 1.27) | 116 per 1,000 | **68 fewer per 1,000** (from 101 fewer to 31 more) |

**CI:** Confidence interval; **RR:** Risk ratio

#### Explanations

a. Downgraded due to serious risk of bias in three studies and moderate risk of bias in four studies

b. Downgraded considering the upper end of 95% CI are close to 1 and the results are not stable on sensitivity analysis

c. Downgraded due to moderate to serious risk of bias in included studies

d. Downgraded due to increased tendency of bleeding noted with DOAC in Inokashi 2021
